# Subclavian Artery Pseudoaneurysm Following Bedside Temporary Hemodialysis Catheter Insertion: A Case Report

**DOI:** 10.3390/medicina59112038

**Published:** 2023-11-19

**Authors:** Sang-Woo Kim, In-Chul Nam, Doo-Ri Kim, Jeong-Jae Kim, Sung-Eun Park

**Affiliations:** 1 Department of Radiology, School of Medicine, Jeju National University, Jeju National University Hospital, 15, Aran 13-gil, Jeju 63241, Republic of Korea; swkim199310@gmail.com (S.-W.K.); antisors@naver.com (D.-R.K.); jsquare8057@gmail.com (J.-J.K.); 2 Department of Radiology, School of Medicine, Gyeongsang National University, Gyeongsang National University Changwon Hospital, 11 Samjeongja-ro, Changwon 51472, Republic of Korea; uneyes@gnuh.co.kr

**Keywords:** temporary hemodialysis catheter, subclavian, pseudoaneurysm, embolization

## Abstract

A pseudoaneurysm of the subclavian artery following central venous catheter placement is a rare but potentially fatal complication that often requires surgical intervention. However, surgical repair of the subclavian artery remains challenging. Herein, we report the case of a male patient undergoing hemodialysis who developed a pseudoaneurysm of the subclavian artery after a bedside central vein catheter placement. Hemostasis was successfully achieved by selecting the pseudoaneurysm using a microcatheter. At the 10-month follow-up, the pseudoaneurysm was completely excluded, and the patient was in a stable condition. The patient underwent native arteriovenous fistula creation and hemodialysis. Endovascular treatment could be an effective nonsurgical treatment for subclavian artery pseudoaneurysms and has been attempted as a first-line treatment option.

## 1. Introduction

Insertion of a central venous catheter (CVC) is a common practice. Catheter insertions at the bedside without imaging assistance are common [1], and such procedures exhibit a relatively high failure rate compared to image-guided catheter insertion owing to anatomical variations of the vein [2]. Additionally, complications such as arterial rupture or pseudoaneurysm can arise during such procedures in the adjacent subclavian artery due to iatrogenic trauma, which can sometimes be dangerous or even fatal [3]. This is primarily attributable to the obscured nature of the subclavian artery by the clavicle, making it difficult to take measures such as proper compression, potentially exacerbating the course of these complications. Herein, we report a case of serious subclavian artery rupture and pseudoaneurysm following temporary hemodialysis (HD) catheter insertion that was successfully treated with coil and N-butyl cyanoacrylate (NBCA) embolization.

## 2. Case Presentation

**Figure 1:**
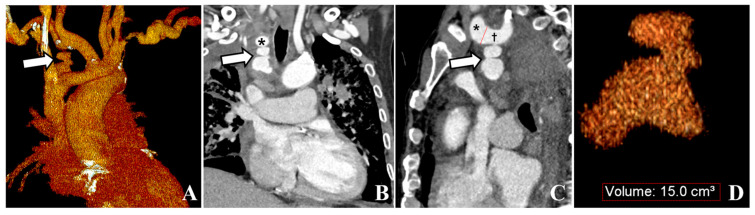
A 77-year-old man shows an irregular-shaped pseudoaneurysm at the most proximal portion of the subclavian artery, extending to the mediastinum. (**A**) The 3D volume rendering image shows a pseudoaneurysm (white arrow). (**B**) The coronal reformatted contrast-enhanced CT demonstrates the pseudoaneurysm (white arrow) extending to the mediastinum. Note the subclavian artery (black asterisk). (**C**) The sagittal reformatted contrast-enhanced CT demonstrates the pseudoaneurysm (white arrow) arising from the subclavian artery (black dagger). Note the common carotid artery (black asterisk). (**D**) The 3D volume rendering image shows an irregular-shaped pseudoaneurysm. The volume of pseudoaneurysm is approximately 15 cc.

**Figure 2:**
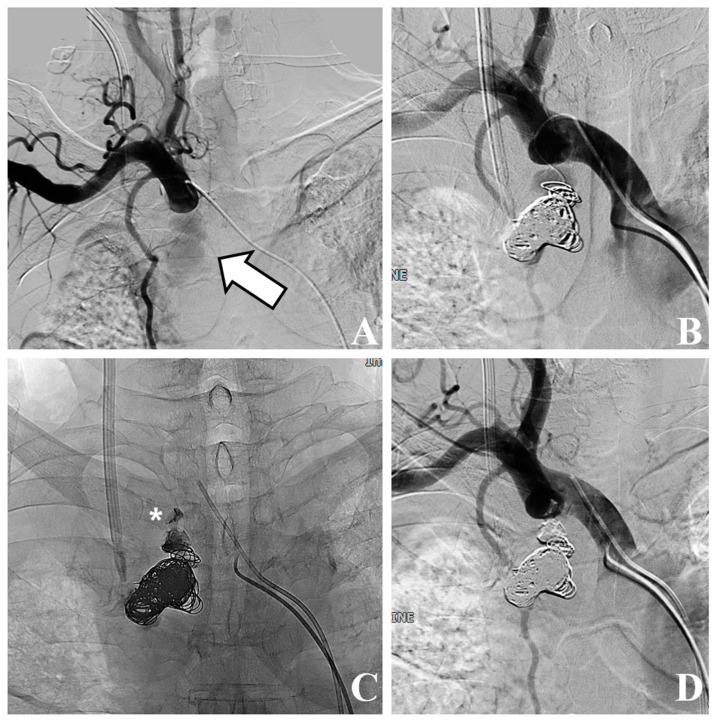
Conventional angiography and coil with N-butyl cyanoacrylate (NBCA) embolization. (**A**) Conventional angiography shows pseudoaneurysm (white arrow) at subclavian artery, extending to mediastinum. (**B**) Embolization is performed using multiple coils. (**C**) Complete embolization is performed using multiple coils and NBCA injection. Note the partial extrusion of NBCA cast (white asterisk) into arterial lumen. (**D**) Final angiography shows patent arterial flow and stable state of extruded cast material.

**Figure 3:**
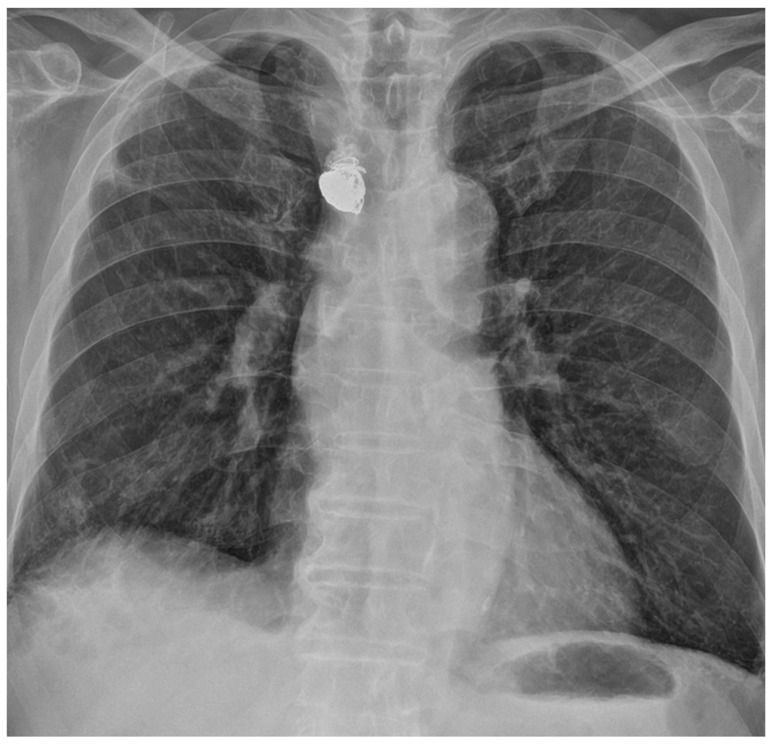
Chest radiographs acquired 10 months after the procedure show stable state of coils and N-butyl cyanoacrylate (NBCA) cast material.

A 68-year-old man with long-standing hypertension and type 2 diabetes mellitus visited our hospital with severe dyspnea. Laboratory tests revealed marked azotemia, with blood urea nitrogen at 87.6 mg/dL and creatinine at 5.5 mg/dL. The patient’s blood pressure was 145/69 mmHg, his heart rate was 92 bpm, and his respiratory rate was 23 cycles/min. A complete blood count showed a white blood cell count of 12.5 × 10^3^/µL and a hemoglobin level of 8.4 g/dL. Chest radiography revealed diffuse increased haziness in both lung fields, increased interstitial marking, blunting of the bilateral costophrenic angles, and cardiomegaly, suggestive of pulmonary edema and bilateral pleural effusion. The attending physician attempted to insert a CVC for temporary HD into the right internal jugular vein without bedside ultrasound guidance. After the procedure, mild swelling at the catheter insertion site prompted a 30 min application of manual compression. Subsequently, no palpable swelling or pulsatile mass was detected, and the patient remained free of any additional complaints, leading to the termination of the procedure. The subsequent day, HD via the same conduit proceeded uneventfully. Owing to an ongoing mild fever that had persisted for the past week, a chest CT scan was conducted for the patient to identify the source of the infection, which revealed a pseudoaneurysm in the right subclavian artery with a mediastinal hematoma (Figure 1). The pseudoaneurysm was located about 1 cm away from the branching point of the subclavian artery and the common carotid artery in the brachiocephalic artery. The neck diameter of the pseudoaneurysm was measured to be 4–5 mm, and although the irregular shape of the pseudoaneurysm made it difficult to determine the exact size or volume, the approximate size was about 4.1 × 2.2 × 3.6 cm in size. The volume of the pseudoaneurysm, estimated using three-dimensional volume rendering image processing, was approximately 15 cc. The attending doctor who performed the CVC insertion at the bedside reported that during the procedure, while inserting the dilator to widen the tract for the catheter, the tip of the dilator was slightly directed towards the medial side rather than following the original vessel course. We hypothesized that the dilator might have injured the subclavian artery during the procedure, possibly because of the tortuous trajectory of the subclavian artery. Given the patient’s overall state and significant concurrent medical conditions, we decided to explore endovascular intervention administered under local anesthesia. Following consultation with the radiologist on duty, the patient was transported to an interventional radiology facility for endovascular treatment. We inserted a 5 Fr vascular sheath through the right common femoral artery to access the subclavian artery. Subsequent angiography revealed a pseudoaneurysm at the most proximal portion of the right subclavian artery, extending to the mediastinum. Stent graft insertion was initially considered. However, owing to the tortuosity of the vessel and the location of the pseudoaneurysm at the proximal part of the subclavian artery, approximately 1 cm distant from the common carotid artery, there was a risk that inserting a stent graft could also occlude the common carotid artery. Consequently, this option was ruled out. Thus, we decided to perform direct embolization of the pseudoaneurysm. Successful selection and localization of the lesion were achieved using a combination of a 5 Fr guiding catheter and a 2 Fr swan-neck microcatheter. Subsequently, embolization of the identified lesion was performed using a combination of multiple coils and NBCA injection (Figure 2). Following the introduction of NBCA into the pseudoaneurysmal sac, the partial extrusion of NBCA casts occurred over several seconds. However, this extrusion did not impede arterial flow, and the extracted adhesive cast remained connected to the interior of the pseudoaneurysmal sac, prompting continued observation of its progression. The procedure was technically successful, with immediate cessation of active extravasation and complete obliteration of the pseudoaneurysm. Four days later, a temporary HD catheter was inserted through the left internal jugular vein under ultrasound and fluoroscopic guidance, and HD was administered for approximately 3 weeks. Subsequently, as continuous HD became necessary, the patient underwent left brachiocephalic native arteriovenous fistula creation and received stable HD without major issues for 10 months (Figure 3).

## 3. Discussion

Bedside CVC insertion is commonly performed in various clinical settings, predominantly in intensive care units and emergency medicine departments, to address the needs of patients requiring immediate medical intervention. Its primary significance lies in its ability to provide reliable access points for applications such as HD, drug administration, and nutritional support. However, the process of catheter insertion has potential intra- and postprocedural complications. These complications can be broadly categorized as early or late. Early complications include hematoma formation, arterial injury, hemo- and pneumothorax injury, and brachial plexus injury, whereas delayed complications include the development of fibroblastic sleeves, catheter-related venous thrombosis, catheter tip migration, and catheter-associated infections [4]. Early complications, particularly those linked to mechanical factors, are of heightened concern because of their significant correlation with increased mortality rates. Complications arising from arterial puncture occur in approximately 2.7–8% of patients undergoing CVC placement [5,6,7]. Risk factors predisposing to the incidence of mechanical complications after bedside central venous catheterization include a low body mass index (BMI; <20 kg/m^2^), limited operator experience, and an increasing number of skin punctures [7].

Localized swelling, palpable pulsations, or hemodynamic instability observed intra- or postprocedurally should raise concerns regarding arterial perforation or related hematomas. If these symptoms are noted, further evaluations, such as ultrasonography, CT angiography, or conventional angiography, are crucial to avoid complications from delayed diagnosis. Once arterial injury secondary to attempting CVC insertion is confirmed, various therapeutic approaches can be considered, including removal of the catheter, external compression, surgical repair, and endovascular treatment [8].

In the case of a subclavian artery pseudoaneurysm, as in the present case, anatomical complexities can impede successful local compression. Additionally, surgical repair can be challenging for managing both the proximal and distal segments of the injured artery. Such repairs often require wide dissection, usually requiring both supra- and infraclavicular approaches and sometimes the use of thoracotomy or median sternotomy [9]. Moreover, many patients undergoing CVC are in a fragile medical state, amplifying the risks associated with general anesthesia or thoracotomy. Therefore, an endovascular strategy may be a feasible alternative for these patients.

Endovascular treatments include the insertion of a covered stent to seal off focal arterial lesions, endovascular compression via prolonged balloon dilatation, direct thrombin injection with or without image guidance, or the embolization of the pseudoaneurysm itself [10,11,12,13]. When deploying a covered stent, the exclusion of arterial structures such as the common carotid artery, vertebral artery, or internal thoracic artery should be avoided [10,11]. In the present case, the pseudoaneurysm was located in the proximal portion of the subclavian artery, which was located about 1 cm away from the common carotid artery. Consequently, using a covered stent carried the risk of occluding the common carotid artery. Additionally, the diameter of the subclavian artery in our patients was 12–13 mm. Considering the tortuosity of the subclavian artery and preventing stent-graft migration, a minimum oversizing of 10–20% was required to sufficiently seal the pseudoaneurysm neck compared to the vessel diameter. This necessitated a stent-graft with a diameter of at least 14 mm, which was not available at our institution at the time of the procedure. In the case of endovascular balloon compression, even with low-pressure balloon tamponade, unexpected arterial dissection due to barotrauma can occur. Additionally, in patients who are not heparinized, prolonged balloon compression might lead to thrombus formation in the distal artery. Such events could potentially cause cerebral infarction or upper extremity ischemia, so prolonged balloon compression was not chosen. With thrombin injection, it is important to monitor the process of injecting thrombin using ultrasound to ensure that the thrombus dose does not enter the arterial circulation. In this patient, ultrasound evaluation was impossible due to the presence of sternum, lung, and costal cartilage on the ventral side. Direct puncture under fluoroscopic guidance or blind puncture for the pseudoaneurysm were also unfeasible. This was due to the lesion’s proximity to critical structures (i.e., left brachiocephalic vein, subclavian artery, carotid artery, etc.), making it extremely challenging to avoid, all while puncturing. Therefore, we opted for a direct embolization of the pseudoaneurysm. Jaldin et al. [12] reported a case report successfully treating subclavian artery pseudoaneurysm near the vertebral artery with direct coil embolization through superselection. Similarly, by employing an appropriate combination of a guiding catheter and microcatheter, we successfully selected the pseudoaneurysm and performed successful embolization using coils and NBCA injection. However, following NBCA injection, a small portion of the NBCA cast inadvertently entered the arterial lumen without impeding arterial flow. Fortunately, at the 10-month follow-up, no embolism was observed, and the patient’s condition remained stable.

## 4. Conclusions

The CVC insertion procedure is relatively safe. However, complications can occur occasionally. Among these, arterial injuries, such as pseudoaneurysms or ruptures, can be fatal. Endovascular treatment of iatrogenic injuries of the subclavian artery can be an alternative to surgical treatment with less morbidity and mortality.

## Data Availability

The dataset generated and/or analyzed during the current study is available from the corresponding author upon reasonable request.
